# Supplementary material for: Characterization of an unusual carlavirus-like RNA from papaya (Carica papaya) lacking essential genes
Source: PLoS One. 2025 Aug 8;20(8):e0329708. doi: 10.1371/journal.pone.0329708 (PMC12334012; doi:10.1371/journal.pone.0329708)
Supplement: S1 Table — Abbreviations: papaya defective virus 1 (PapDfV1), Zhejiang betaflexivirus 2 (ZhBV2), viral construct of papaya defective virus 1 (pLX-AS_D), viral construct of Zhejiang betaflexivirus 2 (pLX-AS_MW), Triple Gene Block 1 (TGB1), and Nucleic Acid Binding Protein (NABP). The expected size in nucleotides (nt) of each fragment is indicated. All primers were designed based on the sequences available in GenBank: OR253703 for PapDfV1, MW897315 for ZhBV2, and MW281334 for the cloning vector pLX-AS. Virus specific primers used for Rapid Amplification of cDNA Ends (RACE) were used with the anchored-oligodT primer provided in 5′/3′ RACE Kit, 2nd Generation (Roche, Germany). (DOCX) [file pone.0329708.s001.docx]

**S1 Table. List of primer sequences used during this study.** Abbreviations: papaya defective virus 1 (PapDfV1), Zhejiang betaflexivirus 2 (ZhBV2), viral construct of papaya defective virus 1 (pLX-AS_D), viral construct of Zhejiang betaflexivirus 2 (pLX-AS_MW), Triple Gene Block 1 (TGB1), and Nucleic Acid Binding Protein (NABP). The expected size in nucleotides (nt) of each fragment is indicated. All primers were designed based on the sequences available in GenBank: OR253703 for PapDfV1, MW897315 for ZhBV2, and MW281334 for the cloning vector pLX-AS. Virus specific primers used for Rapid Amplification of cDNA Ends (RACE) were used with the anchored-oligodT primer provided in 5′/3′ RACE Kit, 2nd Generation (Roche, Germany).

| **Virus** | **Primers F/R (5’ – 3’)** | **Annealing Temp.** | **Amplification size (nt)** | **Application** |
| --- | --- | --- | --- | --- |
| PapDfV1 | TGGCTCTAACCTATAGAACCCC | 57°C | 916 | Amplification of overlapping fragments for genomic resequencing |
|  | CCAAAGGCCACAGCTTCAAAAC |  |  |  |
|  | AAGGTGCTGCTGTTACTTGCG | 57°C | 980 |  |
|  | CTTGTGGTTTTCGGCAGAACC |  |  |  |
|  | CTGATTGGAGATGGTTCATGGC | 57°C | 889 |  |
|  | GCATGCACAACACAAAGGTTC |  |  |  |
|  | GTGGTGGGTGGAAATTGTTGG | 57°C | 845 |  |
|  | GAAGAACCAATTCTGCTGCCC |  |  |  |
|  | CAAACAAATGACTACTGGGGCC | 57°C | 1,002 |  |
|  | CTTGCACCTCCTCAATTTGGCA |  |  |  |
|  | GAGGGGTTCCAAACTAAAGTGG | 57°C | 964 |  |
|  | GAATTAGGTCATTTGGGAGGCC |  |  |  |
|  | TGAGGCTTTTGATGCTTCGC | 57°C | 921 |  |
|  | CCCTTAGAATAAACGCCGTAGC |  |  |  |
|  | GGGAGTTACTTGCTGAGCATAG | 57°C | 272 | RACE 3’ out |
|  | CCTGGTCTCAATCATCCAGGTT | 57°C | 190 | RACE 3’ in |
|  | GCACTCAACTTTTGCTTCGCTT | 57°C | 243 | RACE 5’ out |
|  | GCCTTTGAAGCAATGGCTGATT | 57°C | 156 | RACE 5 in |
|  | GGGAGTTACTTGCTGAGCATAG | 55°C | 226 | Virus detection (TGB1-NABP junction) |
|  | GGTCTGAAGATGCACAAGGACT |  |  |  |
|  | GACTGGGCAGCAAATTTGGGAA | 58°C | 298 |  |
|  | GGTCTGAAGATGCACAAGGACT |  |  |  |
| ZhBV2 | GGGAGTTACTTGCTGAGCATAG | 55°C | 2,352 | Virus detection (TGB1-NABP junction) |
|  | GGTCTGAAGATGCACAAGGACT |  |  |  |
|  | GGTGACCCGCTGCAAAGTTGT | 55°C | 326 | Search of missing regions |
|  | CTTGGCGTGATTGAAATTGCG |  |  |  |
|  | GGTGGCTGTTACAGAGACGG | 55°C | 256 |  |
|  | CTCAGCAGGTTGAATGGCAGC |  |  |  |
| pLX-AS_D/pLX-AS_MW | CTATCCTTCGCAAGACCCTTC | 55°C | 600 | Colony PCR for transformation verification |
|  | TTCAGCGAGGGTTGCTTTATGC |  |  |  |
|  | GGGAGTTACTTGCTGAGCATAG | 55°C | 612 (pLX-AS_D)  2,740 (pLX-AS_MW) |  |
|  | AATCAACAATTCCTGCAGGC |  |  |  |
