# Supplementary material for: Characterization of an unusual carlavirus-like RNA from papaya (Carica papaya) lacking essential genes
Source: PLoS One. 2025 Aug 8;20(8):e0329708. doi: 10.1371/journal.pone.0329708 (PMC12334012; doi:10.1371/journal.pone.0329708)

S1 Figure.
Construct design and agroinoculation methodology. **A)** Schematic representation of the two viral constructs, pLX-AS_MW and pLX-AS_D, designed and tested in this study. Colored arrowed boxes denote open reading frames (ORFs), while black arrowed boxes denote left (LB) or right borders (RB) of the pLX binary vector. Sites corresponding to the 35S promoter from cauliflower mosaic virus (CaMV), the Ribozyme (RiboZ), the NOS terminator, and AarI restriction sites are indicated. **B)** Agroinoculation procedure showing the basic steps applied in this study. A steril toothpick was dipped into the bacterial suspension and was used to puncture the stem, or wet the edge of a cut leaf or a petiole.

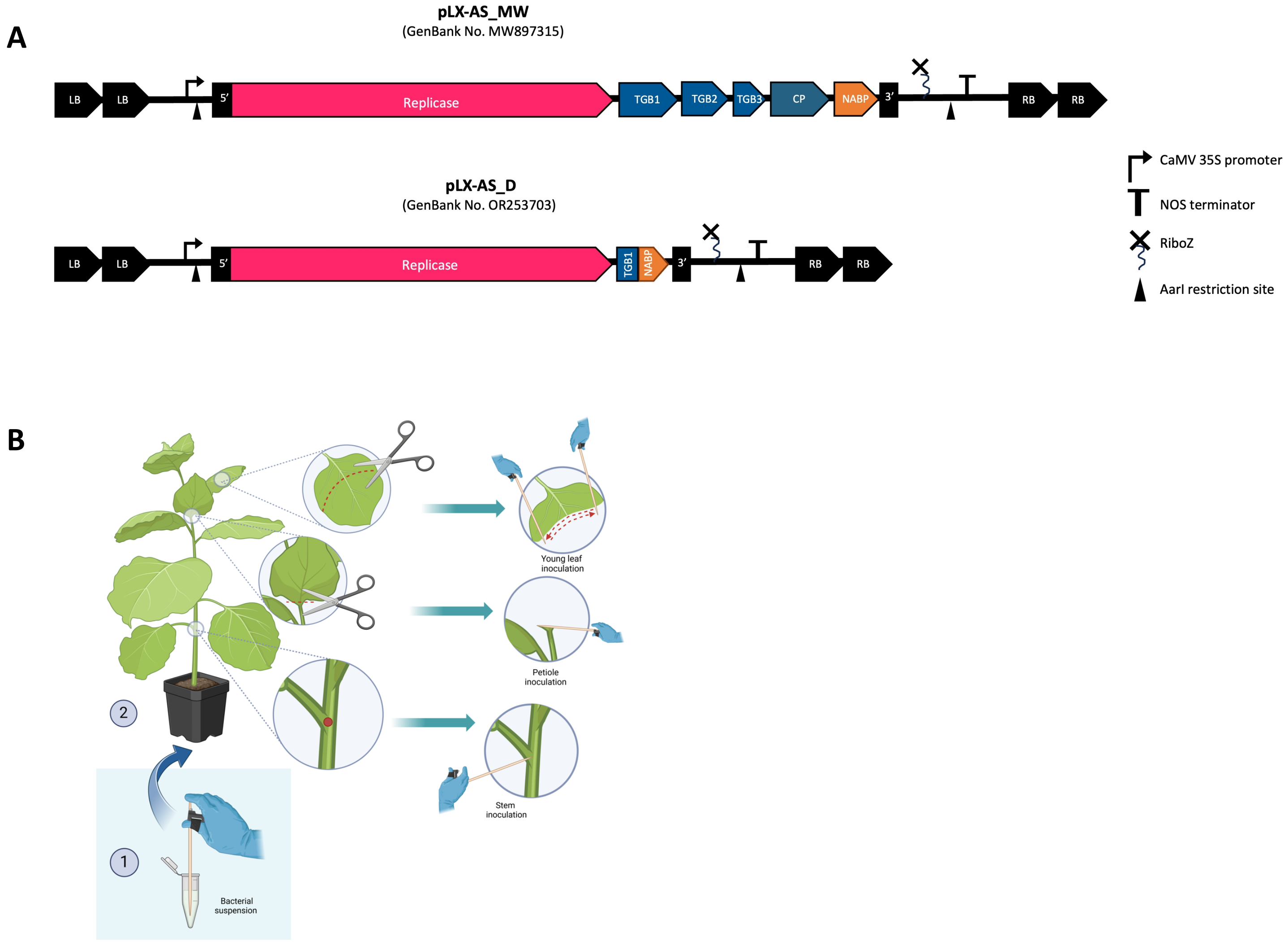

Supplement: S1 Fig — A) Schematic representation of the two viral constructs, pLX-AS_MW and pLX-AS_D, designed and tested in this study. Colored arrowed boxes denote open reading frames (ORFs), while black arrowed boxes denote left (LB) or right borders (RB) of the pLX binary vector. Sites corresponding to the 35S promoter from cauliflower mosaic virus (CaMV), the Ribozyme (RiboZ), the NOS terminator, and AarI restriction sites are indicated. B) Agroinoculation procedure showing the basic steps applied in this study. A steril toothpick was dipped into the bacterial suspension and was used to puncture the stem, or wet the edge of a cut leaf or a petiole. (PDF) [file pone.0329708.s002.pdf]
