# Supplementary material for: Characterization of an unusual carlavirus-like RNA from papaya (Carica papaya) lacking essential genes
Source: PLoS One. 2025 Aug 8;20(8):e0329708. doi: 10.1371/journal.pone.0329708 (PMC12334012; doi:10.1371/journal.pone.0329708)
Supplement: Attachment — Submitted filename: Comment.docx [file pone.0329708.s004.docx]

Comments

Dear Editor,

Please check the following:

Thanks

1. In line 1, the title of the article is clear, attractive, and descriptive, and reflects the overall structure and content of the article

2. This article is written at a good level and the different sections for example introduction, abstract, discussion is readability and having sufficient organization.

3. In the introduction, the importance and position of the research are explained in an acceptable format.

4. In the results section, the data related to the phylogenetic analysis are explained in a suitable manner.

5. In line 38, add a short section on the importance of the discovery of PapDfV1 or other defective viruses in the Abstract and Introduction sections for understanding the evolution of plant viruses

6. In line 66, in the introduction section, provide a reference on possible mechanisms of compensation for deleted genes in carlaviruses or other viruses.

7. In line 226, in the results section, it is better to present a schematic diagram of the genomic structure of PapDfV1 compared to typical carlaviruses. To better understand their differences in terms of genomic structure, based on the figure

8. In line 373, in the conclusion section, further emphasis is placed on the importance of the findings of this research for the direction of future studies.

9. There are a number of sentences in the long text. Please write them in shorter sentences.

10. In line 373, the conclusion title should be written

11. The main headings, including introduction, materials and methods, etc., should be numbered respectively.

12. Please write references to format of journal

13. In total, this article contains a correct writing structure and very desirable content, and with some minor revisions and corrections, it is acceptable.
